# Mesenchymal Stem Cells Reduce the Extracellular Mitochondrial DNA-Mediated TLR9 Activation in Neonatal Hyperoxia-Induced Lung Injury

**DOI:** 10.3390/biomedicines12030686

**Published:** 2024-03-19

**Authors:** Young Eun Kim, So Yoon Ahn, Se In Sung, Misun Yang, Dong Kyung Sung, Won Soon Park, Yun Sil Chang

**Affiliations:** 1 Cell and Gene Therapy Institute, Samsung Medical Center, Seoul 06351, Republic of Korea; ye920.kim@samsung.com (Y.E.K.); soyoon.ahn@samsung.com (S.Y.A.); 2 Department of Health Sciences and Technology, Samsung Advanced Institute for Health Sciences & Technology (SAIHST), Sungkyunkwan University, Seoul 06351, Republic of Korea; 3 Department of Pediatrics, Samsung Medical Center, Sungkyunkwan University School of Medicine, Seoul 06351, Republic of Korea; sein.sung@samsung.com (S.I.S.); misun.yang@samsung.com (M.Y.); dbible@skku.edu (D.K.S.); wonspark@skku.edu (W.S.P.)

**Keywords:** mitochondrial DNA, bronchopulmonary dysplasia, mesenchymal stem cell transplantation, newborn

## Abstract

Mitochondrial DNA (mtDNA) released from dead or injured cells can activate inflammation, and mesenchymal stem cell (MSC) transplantation can reduce inflammation and injury. However, it has not been tested whether the release of mtDNA can be reduced by MSC transplantation. We hypothesized that the level of extracellular mtDNA would be increased after hyperoxia-induced lung injury but reduced after lung injury attenuation by MSC therapy in our newborn rat model. In an in vitro study using a rat lung epithelial L2 cell line, we found that the level of extracellular mtDNA was significantly increased with H_2_O_2_-induced cell death but reduced after MSC co-incubation. In an in vivo study, we confirmed that the levels of cell death, extracellular mtDNA, and inflammatory cytokines were significantly increased in hyperoxic newborn rat lungs but reduced after MSC transplantation. The levels of extracellular mtDNA were significantly and positively correlated with the levels of the inflammatory cytokines. The TLR9/MyD88/NF-κB pathway, which is activated by binding to mtDNA, was also significantly upregulated but downregulated after MSC transplantation. We found a significant positive correlation between inflammatory cytokines and extracellular mtDNA in intubated neonates. The levels of inflammatory cytokines and extracellular mtDNA changed over time in a similar pattern in transtracheal aspirate samples from intubated neonates. In conclusion, increased levels of extracellular mtDNA are associated with increased inflammation in hyperoxia-induced lung injury, and attenuation of lung inflammation by MSC therapy is associated with reduced levels of extracellular mtDNA.

## 1. Introduction

Respiratory support is a lifesaving intervention for ill or premature newborns. However, mechanical ventilation with hyperoxia can cause lung injuries. Hyperoxia-induced lung injury can cause cell damage, cell death, and inflammation. We recently evaluated the efficacy of intratracheal transplantation of mesenchymal stem cells (MSCs), primarily via cell protection and immune modulation, in a hyperoxia-based model of bronchopulmonary dysplasia (BPD) in newborn rats [1,2], and the safety and feasibility of transplanted MSCs in preterm infants at risk of developing BPD in a phase I clinical trial [3].

In injured tissues, damaged cells, such as dying or dead cells, release mitochondrial DNA (mtDNA), known as mitochondrial damage-associated molecular patterns (DAMPs) [4]. In the extracellular space, mtDNA mediates the immune response by activating the mtDNA-binding receptor, toll-like receptor (TLR) 9 [5,6]. Increasing evidence indicates that extracellular mtDNA increases in synovial fluids in arthritis [7], obese plasma [8], severe heart failure [9], and systemic inflammatory response syndrome [10] and that the increased levels of extracellular mtDNA are associated with tissue injury and inflammation [10,11].

However, the role of mtDNA in hyperoxia-induced lung injury and its use as a potential biomarker of lung injury and as a therapeutic target for MSC therapy has not been studied. Thus, in this study, we aimed to evaluate the correlation between extracellular mtDNA and inflammation in the lungs after exposure to hyperoxia and the effect of MSC therapy on attenuating the mtDNA levels and mtDNA-mediated inflammatory reactions via the TLR9-NF-κB pathway.

## 2. Materials and Methods

### 2.1. Mesenchymal Stem Cells

Mesenchymal stem cells (MSCs) were isolated from human umbilical cord blood after neonatal delivery, following an informed consent [1]. The MSCs were cultivated, and their characteristics were confirmed using previously established protocols [1].

### 2.2. In Vitro Model of H_2_O_2_-Induced Lung Injury

The rat lung epithelial L2 cell line was purchased from the Korean Cell Line Bank (Seoul, Republic of Korea). L2 cells were cultured in RPMI 1640 medium (Biowest, Nuaille, France) with 10% fetal bovine serum (Biowest) during cell expansion and passaging, and the cells at 80–90% confluency, the cells were exposed to oxidative stress by incubation with 50, 100, or 200 μM H_2_O_2_ in RPMI 1640 medium. In the MSC study, L2 cells were coincubated with MSCs at a 10:1 ratio. In vitro analyses were performed 2 h after H_2_O_2_ induction.

### 2.3. In-Vitro Cell Survival Assay

Cell viability was measured using the Cell Counting Kit (CCK)-8 assay (Dojindo, Kumamoto, Japan), according to the manufacturer’s instructions. Relative cell viability (%) was determined by normalization to untreated normoxic control cells (100%).

### 2.4. In-Vivo Model of Hyperoxia-Induced Lung Injury

The in vivo experimental protocol was reviewed and approved by the Animal Care and Use Committee of Samsung Biomedical Research Institute (Seoul, Republic of Korea) for studies involving animals, based on detailed justification (IRB File No. 20210208002). The procedures followed the guidelines of the Institutional and National Institutes of Health for Laboratory Animal Care. As previously described [12], timed pregnant Sprague-Dawley rats were purchased from Orient Bio (Gapyeong, Republic of Korea) and housed in individual cages with wood shaving bedding and free access to water and standard chow. The rat pups were spontaneously delivered and reared by dams. Within 10 h of birth, rat pups were randomized into three groups: normoxia control (NC), hyperoxia-induced lung injury control (HC), and hyperoxia-induced lung injury with intratracheal MSC (HM). The rat pups were kept under either normoxia (21% O_2_) or hyperoxia (80% O_2_) from birth until postnatal day 14. For MSC transplantation, rats were anesthetized with an intraperitoneal injection of ketamine and xylazine mixture (45 mg/kg and 8 mg/kg, respectively) and restrained at a fixed angle on a board. MSCs (5 × 10^5^ in 0.05 mL normal saline) were intratracheally transplanted into the MSC–transplanted group using a 26-gauge needle syringe. An equal volume of normal saline was administered to the controls in the same manner as described in a previous study [12]. No operative mortalities or procedure–related complications occurred.

### 2.5. In-Vivo Tissue Preparation

Rat lungs and bronchoalveolar lavage (BAL) fluids were obtained at postnatal day (P) 14 following deep pentobarbital anesthesia (60 mg/kg, i.p.). After transcardiac perfusion with normal ice-cold saline, the muscle around the neck was gently removed to expose the trachea, and a catheter was inserted. To obtain the BAL fluid, 1 mL of phosphate-buffered saline was slowly injected and aspirated several times. This step was repeated two times. The cell–free supernatant of the BAL fluid was prepared by centrifugation at 300× *g* for 15 min at 4 °C without touching the pellet or the bottom of the sample tube. For biochemical analyses, the extracted lung tissues were immediately snap-frozen, stored at −70 °C, and homogenized immediately.

### 2.6. Procurement of Human Samples and Informed Consent

Transtracheal aspirates (TTA) for measurement of extracellular mtDNA and inflammatory cytokine levels were collected after obtaining written parental consent for the TTA sampling from neonates who were intubated for transtracheal aspiration and receiving mechanical ventilation during neonatal intensive care unit (NICU) hospitalization at the Samsung Medical Center, Seoul, Republic of Korea. The study protocol was reviewed and approved by the Institutional Review Board of the Samsung Medical Center for studies involving human samples for detailed justification (IRB File No. 2022-01-116-013). The TTA samples were collected from infants born at the 25th–40th week of gestation during days 1–30 of NICU hospitalization. The TTA samples were transported in an icebox maintained at 4 °C until processing. Upon receipt of the TTA samples within 6 h, the cells from each TTA sample were spun down at 3000× *g* for 10 min, and cell-free supernatants were carefully obtained by avoiding touching any pellets or the inner surface of the sample tubes with pipette tips. Cell-free TTA samples containing extracellular mtDNA were stored at −70 °C prior to experimental use. A total of thirty-one TTA samples were analyzed within two months of collection. The authors had no access to information identifying individual participants during or after data collection. All methods were performed in accordance with the relevant guidelines and regulations for human participants.

### 2.7. DNA Isolation

Extracellular mtDNA in cell culture media, BAL fluids of newborn rats, and cell-free TTA of newborn infants were extracted using the DNeasy Blood & Tissue kit (Qiagen, Hilden, Germany), according to the manufacturer’s protocols. Cell culture media (100 μL), rat BAL fluid (50 μL), rat plasma (100 μL), or human cell-free TTA (50 μL) were used for extracellular DNA isolation, and DNA was eluted in 100 μL of elution buffer. All mtDNA templates were isolated under the same conditions, including the number of cultured cells, volume of the culture medium, and injection volume of PBS, to obtain the BAL fluid. The DNA samples were stored at −20 °C until mtDNA levels were measured.

### 2.8. Measurement of mtDNA Level

The level of mtDNA was measured in both in vitro and in vivo studies, as described previously [13]. Briefly, the level of mtDNA in the cell-free cell L2 culture media and the rat BAL fluid or plasma was measured by conventional polymerase chain reaction (PCR) using AccuPower PCR PreMix (Bioneer, Daejeon, Republic of Korea), and the amplification of rat mtDNA was performed under the following conditions: 5 min hot start at 94 °C, followed by 33 cycles of 94 °C for 30 s, 60 °C for 30 s, 72 °C for 30 s, and a final extension at 72 °C for 5 min. The primer sequences for rat mtDNA were as follows: forward, 5′-AGGACTTAACCAGACCCAAACACG-3′ and reverse, 5′-CCTCTTTTCTGATAGGCGGG-3′. The 770 bp PCR products were resolved and visualized using an E-Gel Power Snap Electrophoresis System (Invitrogen, Carlsbad, CA, USA). The PCR band intensity of rat mtDNA was measured using the ImageJ software (version 1.53k) (National Institutes of Health, Bethesda, MD, USA). The mtDNA levels in human TTA were measured as described by Nakahira et al. [14]. Briefly, the level of mtDNA in the TTA was measured by quantitative PCR (qPCR) using the AccuPower GreenStar qPCR PreMix (Bioneer, Daejeon, Republic of Korea) and the Applied Biosystems Quantstudio 6 Flex Real-Time PCR System (Invitrogen). PCR amplification of human NADH dehydrogenase 1 (ND1, a marker of mtDNA) was performed under the following conditions, as described previously [14,15]: an initiation step for 2 min at 50 °C, followed by a first denaturation step for 10 min at 95 °C, 40 cycles of 95 °C for 15 s and 60 °C for 1 min. The primer sequences for human mtDNA were as follows: forward 5′-ATACCCATGGCCAACCTCCT-3′ and reverse 5′-GGGCCTTTGCGTAGTTGTAT-3′. A standard curve was generated using a human mtDNA plasmid construct (GeneScript NM_173708; ORIGENE, Rockville, MD, USA) in serial dilutions. The ND1 standard curve and the amplification plot of the standard curve are shown in Figure S1. All mtDNA templates were isolated strictly under the same conditions, including the number of cultured cells, the volume of the culture medium, the injection volume of PBS to obtain the BAL fluid, and the volume of DNA elution buffer. Additionally, to accurately quantify the band intensity of mtDNA, PCR and DNA electrophoresis were performed simultaneously on all samples under the same PCR conditions. Experiments were performed in technical duplicate and then averaged by a blinded observer.

### 2.9. Western Blots

The expression levels of the cleaved form of caspase-3, toll-like receptor (TLR) 9, myeloid differentiation primary response (MyD88), and nuclear factor kappa-light-chain-enhancer of activated B cells (NF-κB) in rat lung tissues were measured by western blotting. Rat lung tissues were lysed using the PRO-PREP Protein Extraction Solution (iNtRON Biotechnology, Seongnam, Republic of Korea) and transferred to nitrocellulose membranes. The membrane was blocked and incubated with primary antibodies against cleaved caspase-3 (Cell Signaling Technology, Beverly, MA, USA), TLR9 (Novus Biologicals, Littleton, CO, USA), MyD88 (Santa Cruz Biotechnology, Santa Cruz, CA, USA), NF-κB (Cell Signaling Technology), and glyceraldehyde 3-phosphate dehydrogenase (GAPDH) (Santa Cruz Biotechnology). Western blot was developed by the ECL Prime Western Blotting Detection Reagent (GE Healthcare, Piscataway, NJ, USA) and detected using an Amersham Imager 600 (GE Healthcare Life Sciences, Pittsburg, PA, USA). The detected band intensities were measured using the ImageJ software (version 1.53k) (National Institutes of Health), and the protein of interest/GAPDH ratio was calculated from the band intensities. Experiments were performed in technical duplicate and then averaged by a blinded observer.

### 2.10. Enzyme-Linked Immunosorbent Assay

The levels of inflammatory cytokines, such as interleukin (IL)-1α, IL-1β, and tumor necrosis factor (TNF)-α, in the BAL fluid of newborn rats and in the TTA of newborn infants were measured using commercial enzyme-linked immunosorbent assay (ELISA) kits (R&D Systems, Minneapolis, MN, USA) according to the manufacturer’s protocol. Experiments were performed in technical duplicate and then averaged by a blinded observer.

### 2.11. Statistical Analyses

Data are presented as mean ± standard error of the mean (SEM). Statistical comparisons between the groups were evaluated using one-way analysis of variance (ANOVA) and Tukey’s post hoc analysis. Correlations between the levels of extracellular mtDNA and inflammatory cytokines were analyzed using Spearman’s correlation coefficients. All data were analyzed using the SAS software (version 9.4) (SAS Institute, Cary, NC, USA), and *p*-values < 0.05 were considered statistically significant.

## 3. Results

### 3.1. Extracellular mtDNA Analyses in Rat Lung Epithelial L2 Cells

**Figure 1:**
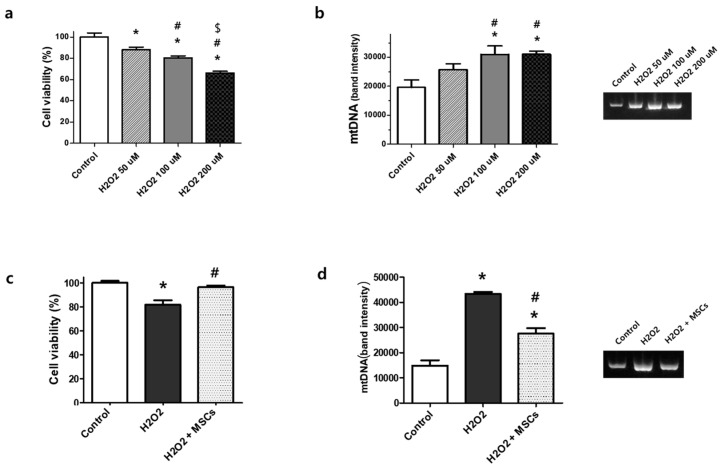
Cell viability and the level of extracellular mtDNA in H_2_O_2_-induced L2 rat lung epithelial cells. (**a**) Cell viability decreased in an H_2_O_2_ concentration-dependent manner in the culture media. (**b**) Densitometric analysis of extracellular mtDNA and the representative PCR gel images. The full-length PCR gel is shown in Figure S2a. Data are expressed as mean ± SEM (*n* = 10 per group). * *p* < 0.05 vs. normoxic control, # *p* < 0.05 vs. H_2_O_2_ 50 μM, $ *p* < 0.05 vs. 100 μM. (**c**) Cell viability of nontreated normoxic control, 100 μM H_2_O_2_ induction, and 100 μM H_2_O_2_ induction with MSC treatment (10:1 ratio). (**d**) Densitometric analysis of extracellular mtDNA and the representative PCR gel image of nontreated normoxic control, 100 μM H_2_O_2_ induction, and 100 μM H_2_O_2_ induction with MSC treatment (10:1 ratio). The full-length PCR gel is shown in Figure S2b. Data are expressed as mean ± SEM (*n* = 10 per group). * *p* < 0.05 vs. normoxic control, # *p* < 0.05 vs. H_2_O_2_.

H_2_O_2_-induced stress significantly reduced the viability of rat lung epithelial L2 cells and increased the levels of extracellular mtDNA in the culture media in an H_2_O_2_ dose-dependent manner (Figure 1a,b). However, H_2_O_2_-induced cell death was significantly improved after MSC co-incubation (Figure 1c), and the level of extracellular mtDNA was significantly reduced in the cell culture media (Figure 1d).

### 3.2. Cell Death and Extracellular mtDNA in Hyperoxia-Exposed Newborn Rats

**Figure 2:**
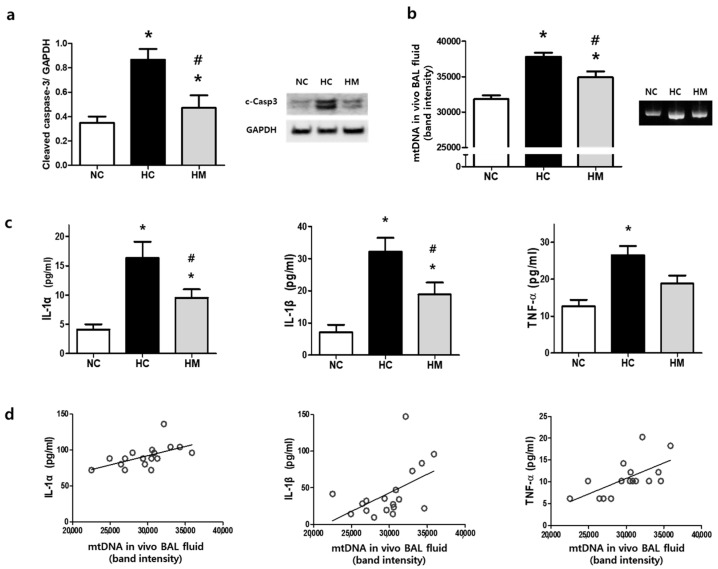
The levels of cell death, extracellular mtDNA, and TLR9/MyD88/NFκ-B in hyperoxic newborn rat lungs. (**a**) Densitometric analysis of cleaved caspase-3 (c-Casp3) levels normalized to glyceraldehyde-3-phosphate dehydrogenase (GAPDH) and the representative western blots of c-Casp-3 and GAPDH (loading control) in rat lung tissues (*n* = 6, 10, and 10 in the NC, HC, and HM groups, respectively). The full-length Western blots are shown in Figure S4a. (**b**) Densitometric analysis of mtDNA levels and the representative PCR gel images of rat BAL fluid (*n* = 12, 14, and 18 in the NC, HC, and HM groups, respectively). The full-length PCR gel is shown in Figure S4b. (**c**) The levels of interleukin (IL)-1α, IL-1β, and tumor necrosis factor (TNF)-α in the newborn rat BAL fluid (*n* = 6, 10, and 10 in the NC, HC, and HM groups, respectively). (**d**) X-axes: level of mtDNA in BAL fluid of HC group of newborn rats (*n* = 18). Y-axes: levels of IL-1α, IL-1β, and TNF-α (pg/mL) in the BAL fluid, respectively (R^2^ = 0.315, *p* < 0.05; R^2^ = 0.248, *p* < 0.05; R^2^ = 0.403, *p* < 0.01). Data are expressed as mean ± SEM. * *p* < 0.05 vs. NC, # *p* < 0.05 vs. HC. NC, normoxic control group; HC, hyperoxic group; HM, hyperoxia with MSC transplantation group.

Hyperoxic lung injury significantly increased lung cell death, marked by an increased level of cleaved caspase-3. However, this increase in cell death in the hyperoxic group was significantly attenuated after MSC transplantation into newborn rat lung tissues (Figure 2a). The level of extracellular mtDNA in BAL fluid was significantly increased after hyperoxic lung injury, but this increase in mtDNA level after hyperoxic lung injury was significantly reduced following MSC transplantation in newborn rats (Figure 2b). The levels of cleaved caspase-3 and extracellular mtDNA were significantly positively correlated in the lungs of newborn rats (Figure S3).

### 3.3. Correlation between the Inflammation and Extracellular mtDNA Levels in Hyperoxia-Exposed Newborn Rats

Hyperoxic lung injury significantly increased the levels of inflammatory cytokines, such as IL-1α, IL-1β, and TNF-α, in the BAL fluid of the hyperoxic group. However, the levels of inflammatory cytokines, which increased after hyperoxic lung injury, were significantly reduced after MSC transplantation (Figure 2c). In the correlation analysis of inflammatory cytokine levels with extracellular mtDNA levels, a significant correlation was observed in the BAL fluid from hyperoxic neonatal rat lungs (Figure 2d). The level of extracellular mtDNA in plasma was significantly increased after hyperoxic lung injury, but this increase in extracellular mtDNA level in plasma after hyperoxic lung injury was significantly reduced, followed by MSC transplantation in newborn rats (Figure S5a). In the plasma, the inflammatory cytokine levels did not increase as much as those in the BAL fluid after hyperoxic lung injury (Figure S5b). The levels of IL-1β in the plasma were significantly higher in the hyperoxic group than in the normoxic control group; however, IL-1α and TNF-α levels in the plasma were not significantly different from those in the normoxic control group. The plasma levels of inflammatory cytokines were not significantly correlated with the extracellular mtDNA levels in the plasma (Figure S5c).

### 3.4. TLR9/MyD88/NF-κB Pathway in Hyperoxia-Exposed Newborn Rats

**Figure 3:**
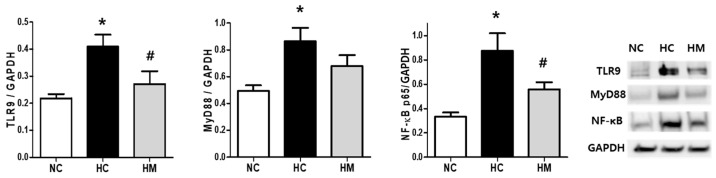
Densitometric analysis of TLR9, MyD88, and NF-κB levels normalized to GAPDH and the representative western blots of TLR9, MyD88, NF-κB, and GAPDH (a loading control) in rat lung tissues. (*n* = 6, 10, and 10 in the NC, HC, and HM groups, respectively). Full-length Western blots are shown in Figure S6. Data are expressed as mean ± SEM. * *p* < 0.05 vs. NC, # *p* < 0.05 vs. HC. NC, normoxic control group; HC, hyperoxic group; HM, hyperoxia with MSC transplantation group.

Hyperoxic lung injury significantly increased the expression levels of TLR9, MyD88, and NF-κB, but the expression of these targets was significantly reduced following MSC transplantation in newborn rat lung tissues (Figure 3).

### 3.5. Extracellular mtDNA Analyses in Transtracheal Aspirates of Human Preterm Infants

**Figure 4:**
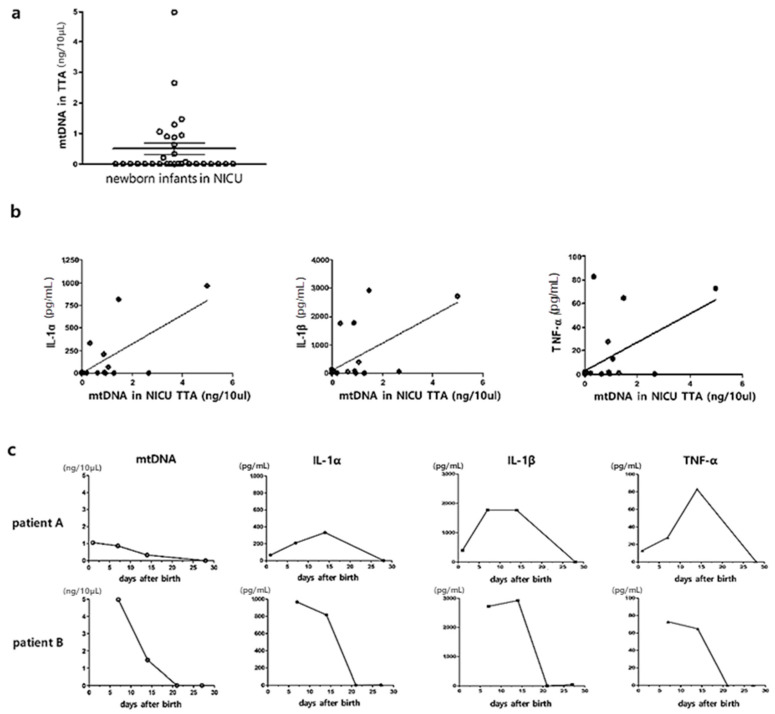
Correlation analyses between the levels of mtDNA and inflammatory cytokines in the TTA of intubated newborn infants hospitalized in the NICU. (**a**) The level of extracellular mtDNA in the TTA of newborn infants (*n* = 31) is plotted on the Y-axis. (**b**) X-axis: level of mtDNA in TTA of newborn infants (*n* = 31). Y-axis: levels of IL-1α, IL-1β, and TNF-α (pg/mL) in the TTA, respectively (R^2^ = 0.532, *p* < 0.001; R^2^ = 0.396, *p* = 0.0001; R^2^ = 0.317, *p* = 0.001, respectively). (**c**) Time series observation of levels of extracellular mtDNA, IL-1α, IL-1β, and TNF-α in TTA of two patients (patient A and B), respectively. X-axis: days after birth. Y-axis: level of mtDNA in the TTA of newborn infants.

A broad range of concentrations of extracellular mtDNA were detected in the TTA of infants requiring mechanical ventilation during NICU hospitalization (Figure 4a). The level of extracellular mtDNA had a significant positive correlation with the levels of inflammatory cytokines such as IL-1α, IL-1β, and TNF-α (Figure 4b). In the TTA time-serially obtained four times, the changes in the levels of extracellular mtDNA were similar to those of the inflammatory cytokines (Figure 4c).

## 4. Discussion

In the present study, we demonstrated that the mtDNA release was significantly increased in H_2_O_2_-induced L2 lung epithelial cells and hyperoxia-induced neonatal lungs, as well as in the TTA of ventilated preterm infants. Additionally, the level of extracellular mtDNA in the BAL fluid of hyperoxia-exposed newborn rats and the TTA of ventilated infants showed a significant positive correlation with the levels of inflammatory cytokines. The potential of mtDNA as a therapeutic target was tested using in vitro and in vivo models of hyperoxia-induced lung injury. Cell death and mtDNA release were significantly increased in H_2_O_2_-induced L2 lung epithelial cells and hyperoxia-treated lungs. However, the increase in cell death and mtDNA release were significantly reduced after MSC transplantation. In an in vivo study, the TLR9/MyD88/NF-κB pathway, activated by binding with mtDNA, and levels of inflammatory cytokines were significantly upregulated in hyperoxia-induced lungs but downregulated after MSC transplantation in a newborn rat model. Our results suggest that the level of extracellular mtDNA in the lung increases with cell death caused by hyperoxic injury, which causes the upregulation of inflammatory cytokines via TLR9. However, MSC transplantation attenuates cell death and extracellular mtDNA levels and, consequently, downregulates inflammatory mediators in hyperoxia-induced neonatal lung injury. The protective role of MSCs in various inflammatory diseases has been previously reported [16]. However, this is the first study demonstrating that MSCs significantly attenuated lung inflammation by reducing mtDNA release and consequently downregulating the TLR9-dependent pathway, as well as showing a significant correlation between lung inflammation and extracellular mtDNA concentration in animal and clinical studies. This suggests that the level of extracellular mtDNA in TTA may be applied as a potential auxiliary biomarker for BPD and attenuation of lung injury after MSC transplantation in preterm infants at risk of developing BPD.

According to previous studies, mtDNA is released into the joint fluids in rheumatoid arthritis [7] and released into circulation in trauma [10], acute respiratory distress syndrome, sepsis, and systemic inflammatory response syndrome, and the increased level of circulating mtDNA is associated with high mortality [14,17]. In this study, we observed a significant positive correlation between cell death and extracellular mtDNA levels, as well as between the extracellular mtDNA and inflammatory cytokine levels in hyperoxia-exposed newborn rat lungs. TLRs are involved in sterile inflammation and their activation causes inflammation [18]. Damaged and dead cells release mtDNA, and TLR9 is the only pattern recognition receptor for mtDNA among TLRs [19]. mtDNA has specific features, such as small, circular, and bacterial-like DNA containing unmethylated CpG motifs, known as mtDNA damage-associated molecular patterns (DAMPs) [20]. Due to its bacterial DNA-like structure, mtDNA triggers TLR9 signaling and stimulates the immune system as an inflammasome activator [21,22]. TLR9, which binds to mtDNA, recruits MyD88, activates NF-κB, and mediates the inflammatory response by upregulating the expression of proinflammatory cytokines [22]. In the previous studies, intratracheal and intravenous injection of mtDNA caused inflammatory cell infiltration into the lung tissues and high production of inflammatory cytokines through the TLR9 dependent pathway [21,23], and TLR9 inhibition significantly attenuated the mtDNA-mediated sitevere lung inflammation [23,24].

Clinical symptoms of BPD occur at a high risk when exposed to frequent ventilation and systemic inflammation in the early neonatal period [25]. Cell-free circulating mtDNA can trigger systemic inflammation, and the blood levels of cell-free circulating mtDNA have been suggested as predictors of systemic inflammation associated with a high mortality rate [9,10,26,27]. In the present study, plasma levels of cell-free extracellular mtDNA were significantly higher in newborn rats with hyperoxic lung injuries than in normoxic controls and were significantly reduced after lung injury attenuation by MSC therapy. This suggests that the levels of circulating extracellular mtDNA may reflect systemic injury in newborn rats with hyperoxia-induced lung injury. However, the plasma levels of inflammatory cytokines in hyperoxia-exposed newborn rats did not significantly increase compared to those in normoxic controls and did not have a significant correlation with the plasma levels of extracellular mtDNA.

## 5. Conclusions

We showed that extracellular mtDNA levels in the BAL fluid and TTA are associated with the severity of hyperoxia-induced lung inflammation and that mtDNA-mediated lung inflammatory reactions via the TLR9-NF-κB pathway were attenuated after MSC therapy. Considering that the level of extracellular mtDNA showed a changing pattern similar to or slightly earlier than that of IL-1α, IL-1β, and TNF-α in the rat BAL fluid and human TTA, our results suggest that the extracellular mtDNA levels in the BAL fluid or TTA may be a candidate biomarker for both the degree of lung injury and lung recovery after treatment. Analysis of extracellular mtDNA levels via quantitative PCR may be an easy and quick method to diagnose hyperoxia-induced lung injury using a small amount of sample. This suggests that the extracellular mtDNA levels in the BAL fluid or TTA could be an auxiliary diagnostic value of lung inflammatory biomarkers, as well as a therapeutic target in hyperoxia-induced lung injury.

## Data Availability

The authors confirm that the data supporting the findings of this study are available within the article and its Supplementary Materials. The raw data that support the findings of this study are available from the corresponding author upon request.

## Supplementary Materials

The following supporting information can be downloaded at https://www.mdpi.com/article/10.3390/biomedicines12030686/s1, Figure S1: The qPCR standard curve for human ND1 gene and the amplification plot of the standard. Figure S2: Full-length PCR gels shown in Figure 1b,d. Figure S3: Correlation analyses of levels of cleaved caspase-3 and mtDNA release. Figure S4: Full-length Western blots shown in Figure 2a and full-length PCR gels shown in Figure 2b. Figure S5: Levels of extracellular mtDNA and inflammatory cytokines measured in plasma of newborn rats. Figure S6: Full-length Western blots shown in Figure 3.
