# Supplementary material for: Mesenchymal Stem Cells Reduce the Extracellular Mitochondrial DNA-Mediated TLR9 Activation in Neonatal Hyperoxia-Induced Lung Injury
Source: Biomedicines. 2024 Mar 19;12(3):686. doi: 10.3390/biomedicines12030686 (PMC10968483; doi:10.3390/biomedicines12030686)
Supplement: Supplementary file 1 [file biomedicines-12-00686-s001.zip › biomedicines-2681835-supplementary.pdf]

Supplementary materials

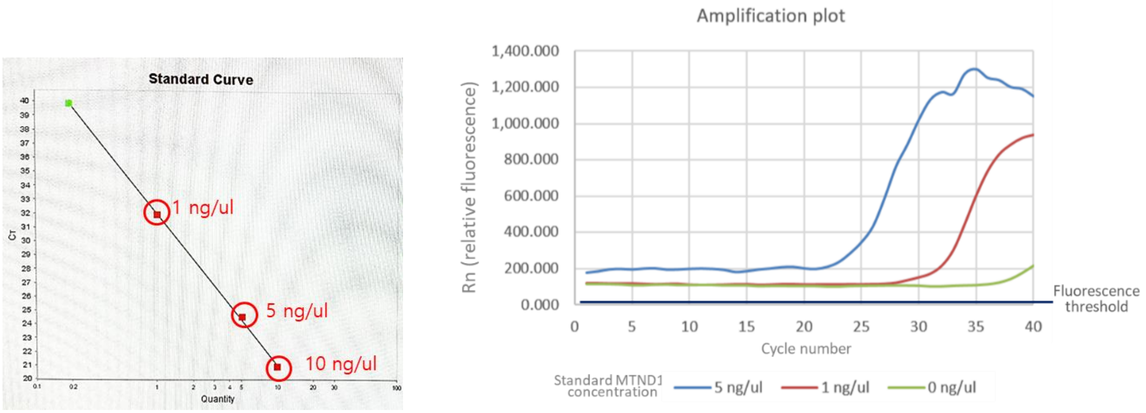

Supplementary Figure S1. The real time PCR standard curve for human MTND1 gene (left panel) and the amplification plot of the standard (right panel), shown in Figure 4.

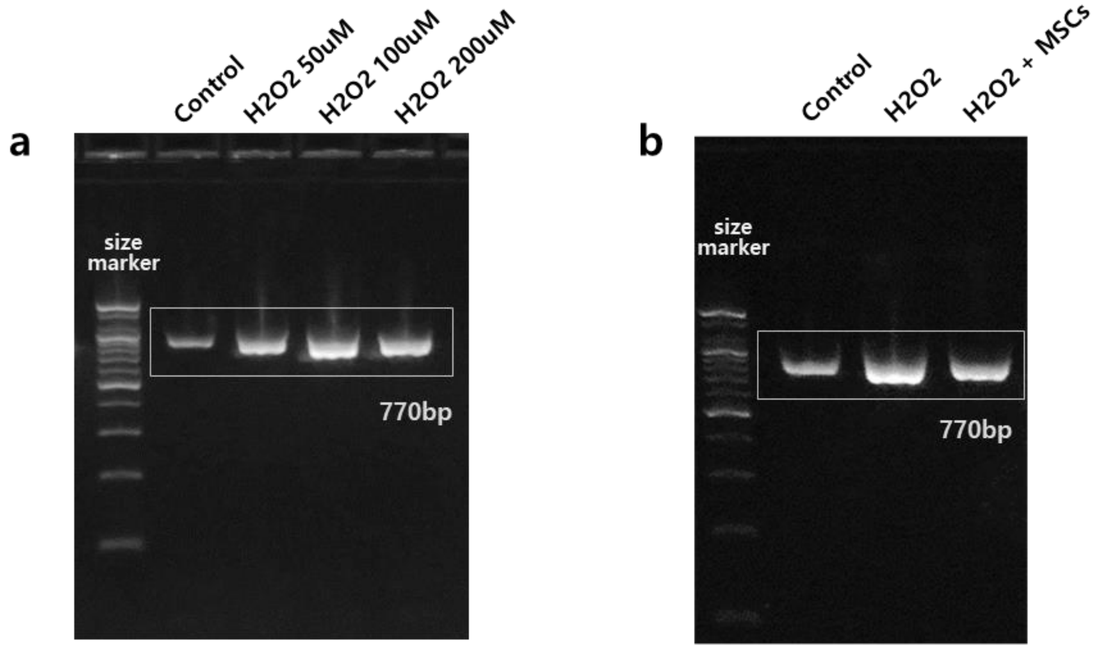

Supplementary Figure S2. Full-length PCR gels shown in Figure 1b and Figure 1d.

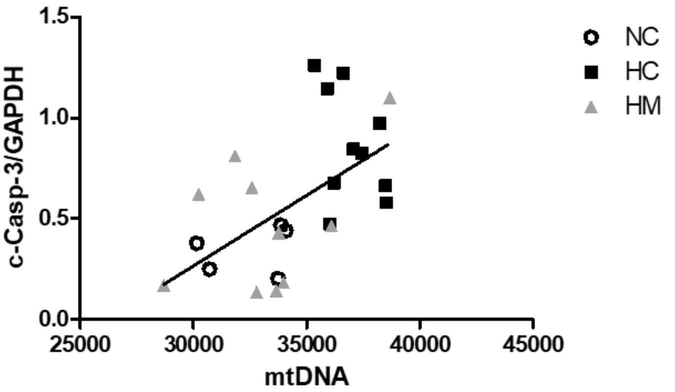

Supplementary Figure S3. Correlation analyses of levels of cleaved caspase-3 (c-Casp-3) and mtDNA release, shown in Figure 3 and b, in hyperoxia-induced lung injury in newborn rats. Y-axis: level of c-Casp-3, normalized to GAPDH, measured in lung tissues. X-axis: level of extracellular mtDNA measured in the BAL fluids (R^2 = 0.314, P < 0.01). (n = 6, 10 and 10 in NC group, HC group and HM group, respectively). NC, normal control group; HC, hyperoxia group; HM, hyperoxia with MSC transplantation group.

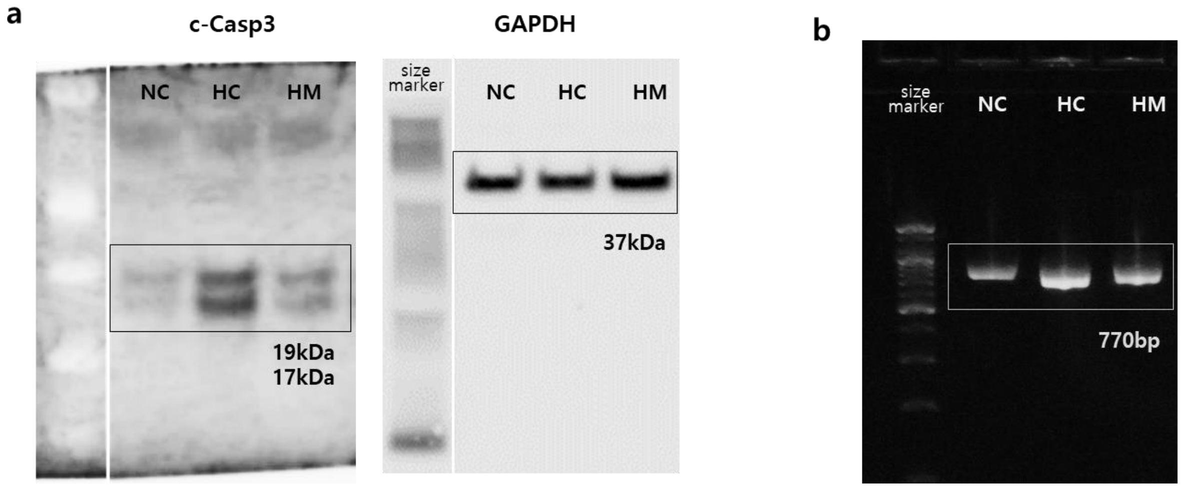

Supplementary Figure S4. Full-length Western blots shown in Figure 2a and full-length PCR gels shown in Figure 2b.

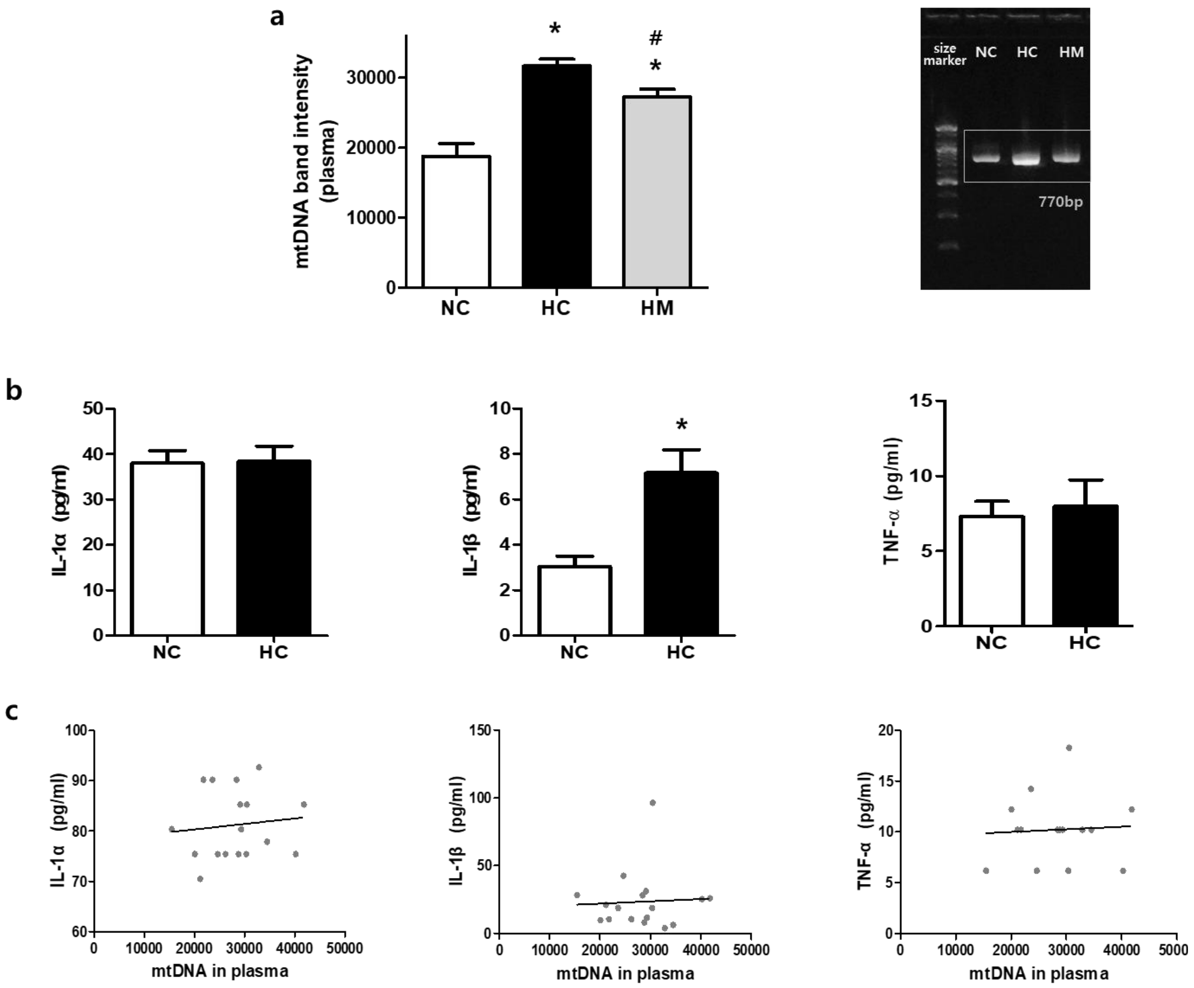

Supplementary Figure S5. Levels of extracellular mtDNA and inflammatory cytokines measured in plasma of newborn rats. (a) Level of extracellular mtDNA in plasma in NC, HC, and HM groups (n = 12, 14 and 18 in NC group, HC group and HM group, respectively). (b) Levels of inflammatory cytokines (IL-1α, IL-1β and TNF-α) in plasma of the HC group compared to the NC group (n = 18/ group) (c) and correlation analyses of levels of extracellular mtDNA and inflammatory cytokines (IL-1α, IL-1β and TNF-α) in hyperoxia-induced lung injury (HC) group (n = 18). Data are given as mean ± SEM. *P < 0.05 vs. NC. #P < 0.05 vs. HC. NC, normal control group; HC, hyperoxia group; HM, hyperoxia with MSC transplantation group.

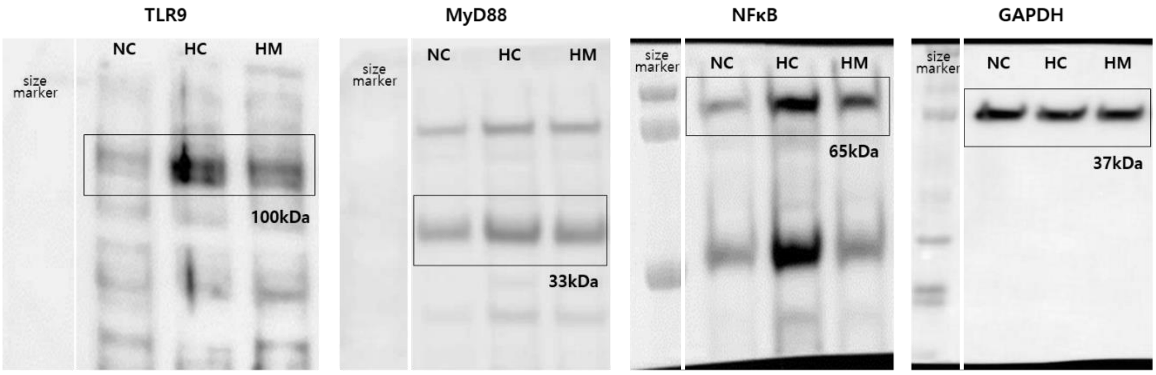

Supplementary Figure S6. Full-length Western blots shown in Figure 3.
